# Predictors of delayed encephalopathy after acute carbon monoxide poisoning: a literature review

**DOI:** 10.3389/fmed.2025.1559264

**Published:** 2025-03-26

**Authors:** Yongjing Wang, Zunzhen Zhou, Dailiang Zhang, Yuan Jiang

**Affiliations:** Clinical Medical College, The First Affiliated Hospital of Chengdu Medical College, Chengdu, Sichuan, China

**Keywords:** carbon monoxide poisoning, DEACMP, predictors, exosomes, miRNAs

## Abstract

Delayed encephalopathy after acute carbon monoxide poisoning (DEACMP) is one of the severe complications that can occur after acute carbon monoxide poisoning (ACOP). The pathogenesis of DEACMP is complex, featuring a delitescence onset and poor prognosis. As a result, many scholars are concentrating on identifying predictors of DEACMP and evaluating their effects, including clinical characteristics, laboratory indicators, neuroelectrophysiology, imaging examination, and genetic susceptibility. However, current identified predictors lack consensus and their clinical application is limited. Therefore, we need to explore new predictors. Exosomes, the smallest extracellular vesicles (EVs) with nano-size, participate in both the physiological and pathological processes of the brain, and the changes in their content can provide valuable information for clinical diagnosis and evaluation of neurodegenerative diseases, suggesting that they may serve as a potential biomarker. However, the practicability of exosomes as biomarkers of DEACMP remains unclear. In the present review, we first introduced the pathogenesis of DEACMP and the currently identified predictors. Then, we also discussed the possibility of exosomes as the biomarkers of DEACMP, aiming to stimulate more attention and discussion on this topic, thereby providing meaningful insights for future research.

## Introduction

Acute carbon monoxide poisoning (ACOP) is a hypoxic disease caused by excessive carbon monoxide (CO) inhalation and is one of the most common causes of poisoning-related deaths worldwide (1). According to statistics, the global cumulative incidence rate of ACOP is 137 cases per million, and mortality is 4.6 deaths per million (2). In many countries, especially in winter, CO exposure increases due to stove heating, coal gas leak from a water heater, or inhalation during a fire, resulting in a higher incidence rate (1–4). CO is a tasteless, colorless, odorless, and nonirritating but highly toxic gas, so the patients may not realize they are poisoned CO unless they experience symptoms such as dizziness, headache, nausea, vomiting, consciousness change, or even coma (5). There have been reports that mortality has significantly declined during the last 25 years due to continued public education, enhanced efficacy of residential CO alarms, and more efficient therapeutic management of patients with CO poisoning (2). However, inhaled CO mainly binds with hemoglobin to form carboxyhemoglobin (COHb), leading to tissue hypoxia, oxidative stress, and inflammation in the heart, brain, nerves, and other tissues after ACOP (3). About 10–30% of surviving patients may develop delayed encephalopathy after acute carbon monoxide poisoning (DEACMP), with symptoms including cognitive dysfunction, personality changes, psychosis, and even Parkinsonism symptoms after a lucid interval (also known as the pseudo-recovery period) ranging from a few days up to months from the ACOP (5, 6).

So far, the pathogenesis of DEACMP is still unclear, involving multiple aspects such as ischemia and hypoxia, oxidative stress injury, reperfusion-oxygenation injury, and so on. As a result, effective treatment options for DEACMP are limited. In addition, the disability rate of DEACMP can be as high as 78%, and the case fatality rate is 31%, causing a heavy burden to families and society (7). DEACMP is characterized by neuronal apoptosis and necrosis, leading to irreversible damage to neural function. Timely identification of DEACMP can help patients receive treatment as early as possible. Scholars have attempted to identify predictors of DEACMP based on clinical characteristics, laboratory indicators, neuroelectrophysiology, imaging examination, and even genetic susceptibility. Although some valuable findings have emerged, current identified predictors lack consensus and their clinical application is limited due to specificity, feasibility, and cost. Therefore, there is a need to explore new predictors for DEACMP.

Mounting evidence indicates that exosomes play a vital role in the pathogenesis of various brain diseases, such as neurodegenerative diseases, ischemic and hemorrhagic stroke, and brain cancer (8–10). Exosomes, a subtype of extracellular vesicles (EVs), are secreted from various cells and transport proteins, lipids, and nucleic acids for intercellular communication. Brain cells can release exosomes to regulate physiological and pathologic processes in the brain (11). The contents of exosomes reflect the physiological and pathological properties of the cell of origin, suggesting that brain cell-derived exosomes may be a source of brain disease biomarkers. Notably, these exosomes can cross the blood-brain barrier (BBB) and circulate in peripheral blood and cerebrospinal fluid, allowing researchers to isolate the exosomes from harvested blood serum and cerebrospinal fluid for brain disease screening, diagnosis, prognosis, and monitoring of therapeutic efficacy. For example, many scholars suggest that peripheral blood neuronal-derived exosomes may be the potential biomarkers for Alzheimer's disease (AD) (12, 13). By comparison, reports on exosomes and DEACMP are scarce, indicating that this area has not yet gained widespread attention. The practicality of exosomes as the biomarkers of DEACMP remains unclear. Therefore, in the present article, we will first introduce the pathogenesis of DEACMP and discuss the advantages and disadvantages of the currently identified predictors. Then, we also discussed the possibility of exosomes as the biomarkers of DEACMP, aiming to stimulate more attention and discussion on this topic, thereby providing meaningful insights for future research.

## Mechanism of DEACMP

DEACMP is a type of encephalopathy that occurs in patients after ACOP. After exposure to excessive CO, brain cells experience a series of changes, including ischemia and hypoxia, reperfusion-oxygenation injury, oxidative stress injury, immune-inflammatory cascade reaction, mitochondrial dysfunction, cell apoptosis and necrosis, neurotransmitter disorder, and microenvironment changes (14). It is hard to interpret the pathogenesis of DEACMP from a single perspective. At present, the following statements are mainly:

### Ischemia and hypoxia

The close combination of CO and hemoglobin can reduce the oxygen-carrying capacity of red blood cells and hinder oxygen release, leading to hypoxia in the body. Notably, neurons in the central nervous system are particularly susceptible to ischemia and hypoxia. Hypoxia can induce cellular injury and death in neurons, impairing neuronal function (15). In addition to neurons, CO can damage myocardial and vascular endothelial cells, leading to reduced cardiac ejection function and increased thrombosis. These injuries worsen ischemia and hypoxia in brain tissues, which may induce or aggravate DEACMP (16).

### Oxygen free radical injury

Excessive exposure to CO can lead to mitochondrial dysfunction, inhibition of mitochondrial respiration, and increased generation of reactive oxygen species (ROS). ROS include superoxide anion radical (O_2_.-), hydroxyl radical (.OH), hydrogen peroxide (H_2_O_2_), singlet oxygen (^1^O_2_), and nitric oxide (NO) (17, 18). High levels of ROS can elevate oxidative stress in brain cells, especially in neurons of the CNS. Central system neurons are rich in polyunsaturated fatty acids (PUFAs), which interact with ROS and can lead to lipid peroxidation, making those neurons more susceptible to the toxic effects of ROS (17). In addition, intracellular hypoxia can lead to increased xanthine oxidase (XO) to generate a large amount of O^2^.-, while O^2^.- can react with NO to form a potentially harmful oxidant peroxynitrite, exacerbating cell injury through oxidative stress (19). NO serves as a messenger molecule and may play some role in neurotransmitter release, neural development, and synaptic plasticity. However, excessive NO may be neurotoxic and significantly harm the function of nerve cells after ACOP (20–22). Moreover, it's worth noting that when hypoxic tissues receive sufficient oxygen again (such as hyperbaric oxygen therapy), high oxygen saturation facilitates ROS production, resulting in reperfusion injury and potentially exacerbating the disease. A new research reveals that ACOP induces iron accumulation in the white matter and ferrostatin-1 can reduce iron and ROS deposition to alleviate ferroptosis (23). Ferroptosis can cause lipid peroxidation and a large amount of ROS, leading to cell death. Therefore, the role of ferroptosis in the pathogenesis of DEACMP needs more attention and investagtie in the future.

### Immune injury and inflammatory factors

ACOP can cause inflammatory reactions and immune damage in brain tissue, resulting in increased significant levels of interleukin-4 (IL-4), IL-6, IL-13, and tumor necrosis factor- α (TNF - α) in the blood or cerebrospinal fluid (CSF) of patients after ACOP (24–26, 125). In contrast, brain tissues of CO-poisoned rats has a high interferon-gamma (IFN-γ) level (27). These inflammatory factors play a crucial role in triggering the immune response. Myelin basic protein (MBP), a major myelin protein of the CNS, is synthesized by oligodendrocytes and neuromembrane cells, and its levels can reflect the extent of damage to CNS, making it represent a specific biochemical indicator of CNS damage and acute demyelination (28). Some studies have reported that MBP levels significantly increase in the CSF of ACOP patients (29, 30). CO-mediated oxidative stress induces changes in MBP and promotes the formation of MDA-MBP adducts between MBP and the product of lipid peroxidation malondialdehyde (MDA), resulting in a triggered immunological cascade. In the brain tissue of CO-poisoned rats, lymphocytes exhibited an auto-reactive proliferative response to MBP and a significantly increased number of activated microglia (31). In particular, M1 phenotype microglia can cause neuronal damage due to excessive immune responses by secreting proteolytic enzymes, reactive oxygen species (ROS), and inflammatory cytokines (32, 33).

### Apoptosis and autophagy

Scholars observed neuronal apoptosis in the cerebral cortex, parahippocampal gyrus, and other brain regions, leading to the surviving rats after ACOP exhibited clinical symptoms similar to those of DEACMP patients, such as defects in cognition, learning, and memory (34, 35). When nerve cells are exposed to CO, their mitochondrial membrane potential will decline and release cytochrome c into the cytoplasm, activating the mitochondrial apoptosis pathway (36). In addition, CO can increase intracellular caspase-8 levels to activate the death receptor apoptosis pathway (37). CO-induced apoptosis may be the reason of the decline in the number of type 1 and type 2a neural precursor cells, microglial cells, and oligodendrocyte precursor cells in rats after CO exposure. Ochi et al. suggested that this change in glial cell mount can induce the decrease in hippocampal neurogenesis, leading to cognitive impairment in a rat model of delayed CO encephalopathy (38). In addition to apoptosis, tissue hypoxia would trigger autophagy (39, 40). Autophagy may act as an endogenous protective mechanism against hypoxic/ischemic injury, contributing to endogenous neuroprotective and neuro-recovery (41, 42). CO can promote autophagy in the cytoplasm of exposed cells through the increased generation of mitochondrial ROS to activate HIF-1, p53, FOXO3, and NRF2 for triggering the transcription of BNIP3 and NIX, TIGAR, LC3, and BNIP3 and p62, respectively. Low concentrations of CO-induced autophagy can inhibit cell death and inflammation, exhibiting protective actions (43–46). However, excessive autophagy could lead to the degradation of organelles by lysosomes, resulting in brain cell damage or even cell death (47, 126). Some scholars have suggested that apoptosis and autophagy can be activated by the same upstream signal and are interconnected through various crosstalk mechanisms (48, 49), leading to the coexistence of apoptosis and autophagy in the CO-exposed brain cells. Although the mechanism of transformation and coordination remains unclear, autophagy and apoptosis play crucial roles in the pathogenesis of DEACMP.

### Excitotoxicity

Pathological overactivation of excitatory neurotransmission can cause excitotoxicity, leading to synaptic and neuronal degeneration. Excitotoxicity is one of the primary mechanisms of cell death in the central and peripheral nervous systems. It is now regarded that the elevated Ca^2+^ influx induced by glutamatergic synaptic transmission facilitates the accumulation of Ca^2+^ inside the cell through Ca^2+^-permeable (CP)-AMPA receptors (AMPARs), a critical key factor in causing excitotoxicity (50). For instance, the upregulation of CP-AMPARs triggers the Ca^2+^ influx excitotoxic signaling pathway, resulting in mitochondrial injury, endoplasmic reticulum (ER) stress, activation of apoptotic cascades, and even alterations in the ubiquitin-proteasome system, ultimately causing neuronal cell death. In cases of brain damage caused by hypoxia and ischemia, excitotoxicity causes neuronal cell death, contributing to neurodegeneration and detrimental neurological defects (51). In addition, Ochi et al. found that the mRNA expression of nicotinic acetylcholine receptor (nAChR). Chrna3 was significantly decreased in the hippocampal tissue, while cerebellar Chrna7 expression was significantly increased in the delayed CO encephalopathy rat model, indicating that the change of nicotinic acetylcholine receptors (nAChRs) may affect the cognitive status and play a role in the pathogenesis of DEACMP (52).

## Identified predictors of DEACMP

So far, scholars have identified several valuable predictors for predicting DEACMP. These predictors originate from multiple research fields, including clinical characteristics, laboratory indicators, neuro-electrophysiology, imaging examination, and genetic susceptibility (Table 1). They may assist physicians in assessing the risk of developing DEACMP.

**Table 1:** List of the identified predictors of DEACMP.

**Research fields**	**Identified predictors**	**References**
Clinical characteristics	CO exposure duration > 5 h, History of hypertension	(53)
	Pathological neurological examination	(54)
	Age > 40.5 years, CO exposure duration > 4.8 h, GCS score on-site	(55)
	GCS score < 9	(56)
	CO exposure duration >6 hours, GCS score < 9, seizures, systolic blood pressure < 90 mmHg	(57)
	Age, Source of CO, GCS score, HBOT	(127)
	GCS score ≤ 13	(58)
	Age >50 years, GCS score ≤ 12, History of shock, no use of HBOT	(59)
Laboratory indicators	Increased IL-6 in CSF	(24)
	Increased serum S100B, NSE, and GFAP	(60)
	Increased serum S100B and NSE	(61, 62)
	Serum S100B > 0.165 μg/L	(63)
	Serum S100B > 0.67 μg/L	(64)
	Increased serum NSE	(65)
	Serum NSE level of >20.98 ng/mL at 48 h after presentation at hospital	(66)
	Increased serum S100B and GFAP	(67)
	Increased MBP in CSF	(24)
	Decreased serum netrin-1	(68)
	Plasma copeptin >40.5 pmol/L	(69)
Neuro electrophysiology	SEP, VEP, and BAEP	(70)
Imaging examination	Computed tomography	(71)
	Magnetic resonance imaging	(72)
Genetic susceptibility	NSE gene SNPs (rs2071419 and rs3213434)	(73)
	LRCH1 gene polymorphisms (rs9534475)	(74)
	MBP SNPs (rs764529994)	(75)
	PARK2 SNPs (rs1784594)	(125)
	MBP 5′-side TGGA n gene polymorphism	(76)

### Clinical characteristics

Some retrospective analyses have identified several clinical characteristics as independent predictors of DECAMP, including age (>45 years), CO exposure duration, Glasgow coma scale (GCS) score ( ≤ 9 points), pathological neurological examination, the timing of starting hyperbaric oxygen therapy (HBOT), source of CO, and a history of hypertension and seizures [51–55; Mu et al., (127)]. Among these predictors, age, CO exposure duration, and GCS score are often mentioned in many studies. Mu et al. observed that older patients are vulnerable to DECAMP, and suggested that cardiopulmonary function declines in older patients increase the comorbidities after ACOP (127). Zhang et al. suggested that the tolerance of nerve tissue to hypoxia is weakened in the elderly population, and their gradually degraded central nervous system function will aggravate the condition of DEACMP patients (77). A longer duration of CO exposure means that inhaled CO can cause severe brain tissue hypoxia. The cerebral cortex, white matter, basal ganglia, and the globus pallidus, are the most affected areas in brain tissue. Some scholars considered that patients who are exposed to CO for >5 h have a 1.7 times higher risk of DEACMP compared to those exposed for <5 h (53). GCS score can reflect the degree of consciousness impairment in patients, especially those with traumatic brain injury (TBI). The lower scores indicate a severe coma state and a higher risk for future complications in patients. Many retrospective studies confirmed that lower GCS scores are associated with the occurrence of neurological sequelae in ACOP patients. Some scholars suggested that GCS score is a sensitive, specific, rapid, and valuable parameter in case of assessment on-site compared with that evaluated at the emergency room (55, 78). Recently, Kim et al. developed and tested a clinical scoring system (COGAS), which incorporates five factors associated with poor neurocognitive outcome: age, GCS score, serum creatine kinase (CK), hyperbaric oxygen therapy, and shock. This prediction model has demonstrated excellent discrimination performance for DECAMP and may offer significant clinical predictive value (59). However, most of the identified clinical characteristics are from retrospective studies. These studies have several limitations, such as small sample size, selection or information bias, and incomplete medical records, that can influence the sensitivity and specificity of specific clinical characteristics using logistic regression analysis, resulting in inconsistent conclusions regarding the predictive value in different studies (50, 56). Therefore, current identified clinical characteristics need validation through larger sample sizes and multicenter studies in various populations and regions.

### Laboratory indicators

Interleukin-6 (IL-6) is a pro-inflammatory cytokine involved in chronic inflammation and auto-immune disorders. Previous studies have confirmed significantly increased IL-6 levels in the serum and CSF of patients with ACOP. Meanwhile, the level of IL-6 in CSF may be a predictor of DECAMP rather than the serum IL-6 level (24, 26, 30). Ide et al. suggested that the changes in IL-6 levels in CSF may reflect the degree of demyelination in the cerebral white matter in the early phase of ACOP. The high IL-6 levels in CSF will return to normal, so early CSF sampling is crucial for predicting DECAMP (24). However, lumbar puncture is a relatively invasive technique for CSF sampling. Scholars need to consider its invasiveness and operational risk in the clinical application of measuring IL-6 levels in CSF.

S100B is a calcium-binding protein produced primarily by astrocytes in the CNS. A high level of S100B promotes the expression of inducible nitric oxide synthase (NOS) or pro-inflammatory cytokines, resulting in cytotoxicity to neurons in some neurodegenerative disorders (79, 80). S100B can be measured in CSF and blood and its concentration can reflect the severity of pathogenetic conditions and brain injury prognosis (60, 81). S100B in the blood of patients with ACOP also increased significantly, especially in patients with severe poisoning or suffering from DEACMP (61, 63). Previous studies have shown that S100B can reflect the early neurotoxicity of CO poisoning and is related to the degree of consciousness damage. Therefore, it can serve as a predictor of the development of DEACMP with high sensitivity and specificity (64, 82). However, some scholars suggested that the existing evidence makes it difficult to establish a clear correlation between the severity of brain disease and concentrations of S100B in CSF or serum. In addition, the extra-astrocytic and extracerebral expression of S100B involves various potential confounding factors that may induce a lack of disease specificity in predicting neurodegenerative disorders (83–85).

Neuron-specific enolase (NSE), a cell-specific isoenzyme of the glycolic enolase in nerve and neuroendocrine tissue, is involved in human glucose metabolism. NSE exhibits high activity in brain tissue and cells and is released when axons are injured. In contrast, it is lower in non-neural tissues, serum, and spinal fluid. The detection of NSE can provide a quantitative measurement of neuronal damage, reflecting the extent of primary injury to the brain and the progression of secondary damage (86). Many studies have found significantly elevated serum levels of NSE in patients after ACOP, suggesting that serum NSE could be an effective predictor of DEACMP in the early stage with better sensitivity than S100B. Notably, the detection of serum NSE enhances the prediction accuracy of initial GCS and the diagnosis accuracy for DEACMP (62, 65, 66, 87). However, scholars still are unable to confirm a clear correlation between serum NSE levels and the risk of DECAMP due to the change in serum NSE levels are also closely linked to other conditions such as neuroendocrine tumor (NET), small cell lung cancer (SCLC), melanoma and other diseases (88–90). Therefore, further large-scale studies are necessary to confirm the specificity of serum NSE as an independent factor for predicting DECAMP.

Other studies have reported several predictors for predicting DEACMP, such as glial fibrillary acid protein (GFAP), netrin-1, BMP, and plasma copeptin (67–69, 91). However, these laboratory indicators are easily influenced by the physiological and pathological conditions of the patient's body, leading to the correlation between them and DECAMP also requires further experimental discussion.

### Neuro electrophysiology

Electroencephalography (EEG) is a simple, non-invasive, and low-cost neurophysiological detection method for reflecting the functional status of the nervous system (92). Because of the persistent injury of the cerebral cortex and subcortical area, DEACMP patients' EEG may have irregular low to medium amplitude θ Wave or high amplitude δ Wave and other abnormal EEG in the pseudo-recovery period, even if they have no clinical manifestation. The abnormal rate of EEG has a positive correlation with the severity of DEACMP. By dynamically observing the EEG of patients with CO poisoning, we can gain timely insights into their brain damage and then predict whether they have DEACMP.

Brain-evoked potential (BEP) monitoring can reflect the brain function by detecting the electrical signals generated by specific stimuli (e.g., sound, light, etc.), which includes visual evoked potential (VEP), somatosensory evoked potentials (SEP), brain stem auditory evoked potentials (BAEP), etc. BAEP can sensitively evaluate the degree of brainstem functional injury. The demyelinating lesions caused by DEACMP can cause damage to brainstem function. Abnormal changes in peak and inter-peak latency of BAEP exist in patients with DEACMP. He et al. suggested that multimodality-evoked potentials based on VEP, SEP, and BAEP are sensitive indicators for evaluating brain dysfunction and predicting DEACMP in patients with ACOP (70, 93).

However, EEG or evoked potential results require expertise from professionals. Different professionals may have their perspectives on the results of multiple tests, resulting in inconsistencies in the findings. In addition, few studies have been concerned with using neurophysiological detection methods to predict DEACMP in recent years, leading to a lack of specific evaluation criteria for predicting DEACMP.

### Imaging examination

DEACMP is one uncommon subtype of acquired leukoencephalopathy with typical pathological changes, including symmetrical softening of globus pallidus, generalized or focal degeneration and necrosis of the cerebral cortex, and generalized demyelination of white matter. The low-density area of globus pallidus is the most commonly affected site of hypoxia after ACOP, especially the bilateral symmetric lesions, which are significant for diagnosing DEACMP. In addition, other affected regions, such as the hippocampus, cerebellum, and substantia nigra, also exhibit characteristic changes. Imaging examination is a crucial method for assessing the central nervous system, and scholars have found valuable information in AOCP patients' computed tomography (CT) or magnetic resonance imaging (MRI) scans.

The head computed tomography (CT) scan can reveal diffuse low-density lesions in bilateral white matter and globus pallidus, while Gray-matter—white-matter ratio (GWR) and the ratio of gray matter attenuation to white matter attenuation can be used to predict poor neurological outcomes in patients with hypoxic-ischemic encephalopathy. Wang et al. suggested that GWR-basal ganglia could serve as an indicator for predicting DEACMP with high sensitivity (93.8%) and high specificity (68.7%) (94). Du et al. have reported a brain-integrated CT score designed to identify DEACMP. The patient underwent a brain CT scan within 24 h of admission. Three experienced radiologists calculated the integrated CT score based on the characteristics of the regions of abnormal density, such as the distribution of lesions for each brain region, extent of the lesion, lesion severity, swelling of parenchyma, and others. The integrated CT score can assess the pathologic changes in the brain to semi-quantify lesion severity and may help predict the risks of developing DEACMP. Notably, the integrated CT score could be suitable for application in various medical institutions (95). However, some scholars suggest that CT's sensitivity to recognize acute ischemic lesions is relatively low, limiting its effectiveness in detecting early pathological changes of DEACMP (71).

In contrast to traditional head CT, brain MRI offers high resolution and better recognition ability for intracranial soft tissue and does not involve radiation exposure (96). Brain MRI can effectively detect the early pathological changes associated with DEACMP, such as long T1 and long T2 signals in the bilateral white matter and symmetrical globus pallidus, as well as extensive demyelination of white matter (97, 98). A recent systematic review and meta-analysis reported that brain MRI is a good predictor for DEACMP with 72% sensitivity and 80% specificity (72). Ahn et al. suggested that using MRI to detect abnormal brain damage within 72 h after ACOP patients arrive at the emergency department may assist in predicting DEACMP (71). The conventional head MRI only reflects the changes in the tissue and cell structure of the brain area and has a poor display effect of micro-structural lesions. At present, the application of diffusion-weighted imaging (DWI), diffusion tensor imaging (DTI), and diffusion kurtosis imaging (DKI) have overcome this shortcoming. DWI is a new-generation MRI based on the translational movement of water. It can detect early changes in cerebral ischemia and is sensitive to cytotoxic or vasogenic edema. The apparent diffusion coefficient (ADC) value can quantify the movement of different water molecules at the microscopic level. After ACOP, cerebral hypoxia can cause cytotoxic brain edema, which limits the movement and diffusion of water molecules, resulting in decreased ADC values and relatively increased DWI signals. Therefore, DWI can reflect the typical pathological changes of DEACMP more sensitively and earlier than conventional MRI (99). DTI can show the directionality of water molecule diffusion, and low fractional anisotropy (FA) values can sensitively reflect the slight pathologic changes from progressive demyelination in patients after ACOP, making DTI more suitable than DWI for predicting DEACMP (99). Indeed, pathological changes of DEACMP in the acute phase are too slight to observe using the conventional MRI and DWI or DTI in most cases (100), while DKI can be preferred. DKI is a straightforward extension of the DTI with greater sensitivity to detect damages to the microstructure of the brain than DWI or DTI. Even if there are no abnormal lesions in the brain white and gray matter after ACOP, the high mean kurtosis values of DKI may indicate a poor prognosis (71).

In short, brain CT and MRI all exhibited good application value in the research of DECAMP prediction (101, 102, 128). However, from the perspectives of equipment, cost, and patient compliance, medical institutions may face limitations in utilizing serial imaging examinations to predict DEACMP.

### Genetic susceptibility

The influence of toxic substances on human DNA maps has attracted many scholars' attention. ACOP can induce acute DNA changes in the globus pallidus of the brain, which may also be genotoxic (128). Oztürk et al. suggested that the mutagenic compounds found in coal or wood smoke and ash, as well as hypoxic and reperfusion injury, may primarily contribute to the individual genotoxic of carbon monoxide exposure (103). In recent years, more and more studies have found that the polymorphism of some genes is closely related to the genetic susceptibility of DEACMP, indicating that these genes are susceptibility sites of DEACMP. Mutations in their expression could raise the risk of DEACMP and may act as potential predictors. Xu et al. conducted a screening of 6 NSE single nucleotide polymorphisms (SNPs) and compared the genotype frequencies and alleles of the 6 NSE SNPs (rs2071074, rs2071417, rs2071419, rs11064464, rs11064465, and rs3213434) by different genetic models. They found significant differences in the genotypes and allele frequencies of rs2071419 and rs3213434 in the DEACMP patients and ACOP patients, suggesting that rs2071419 and rs3213434 may be susceptible sites of DEACMP. They speculated that the C allele of the rs2071419 polymorphism and the T allele of the rs3213434 polymorphism in NSE could increase the risk of developing DEACMP, serving as potential risk factors for DEACMP (58). Gu et al. found that four leucine-rich repeats and calponin homology domain containing 1 (LRCH1) gene polymorphisms have a significant association with DEACMP, such as rs1539177 (G/A), rs17068697 (G/A), rs9534475 (A/C), and rs2236592 (T/C). Notably, the allelic A of rs9534475 polymorphism in LRCH1 may provide good early prognostic value for DEACMP (73). Zhang et al. investigated the association of MBP SNPs (rs470555, rs470724, rs4890785, rs595997, rs76452994, and rs921336) with DEACMP. They found that the MBP rs76452994 and rs921366 polymorphisms were associated with DEACMP, especially the G allele of rs76452994 and T allele of rs921336, which may serve as risk factors for DEACMP (74). Other studies have also found that the genetic susceptibility of some gene polymorphisms in DECAMP can vary between sexes. For example, Liang et al. suggested that the allelic variant of PARK2 SNPs rs1784594 is a risk factor for DECAMP, especially in females (75). In contrast, Li et al. (125) found that the allele L of MBP 5′-side TGGA n gene could increase the risk of developing DECAMP in male patients. In addition, some scholars believe that DEACMP results from the combined effects of environmental and genetic factors (104). Exploring genetic susceptibility-related gene loci offers a new perspective for predicting DECAMP. However, only focusing on the susceptibility genes of a specific ethnic group within a specific region may lead to inconsistent research results.

## Exosomes and DEACMP

**Figure 1:**
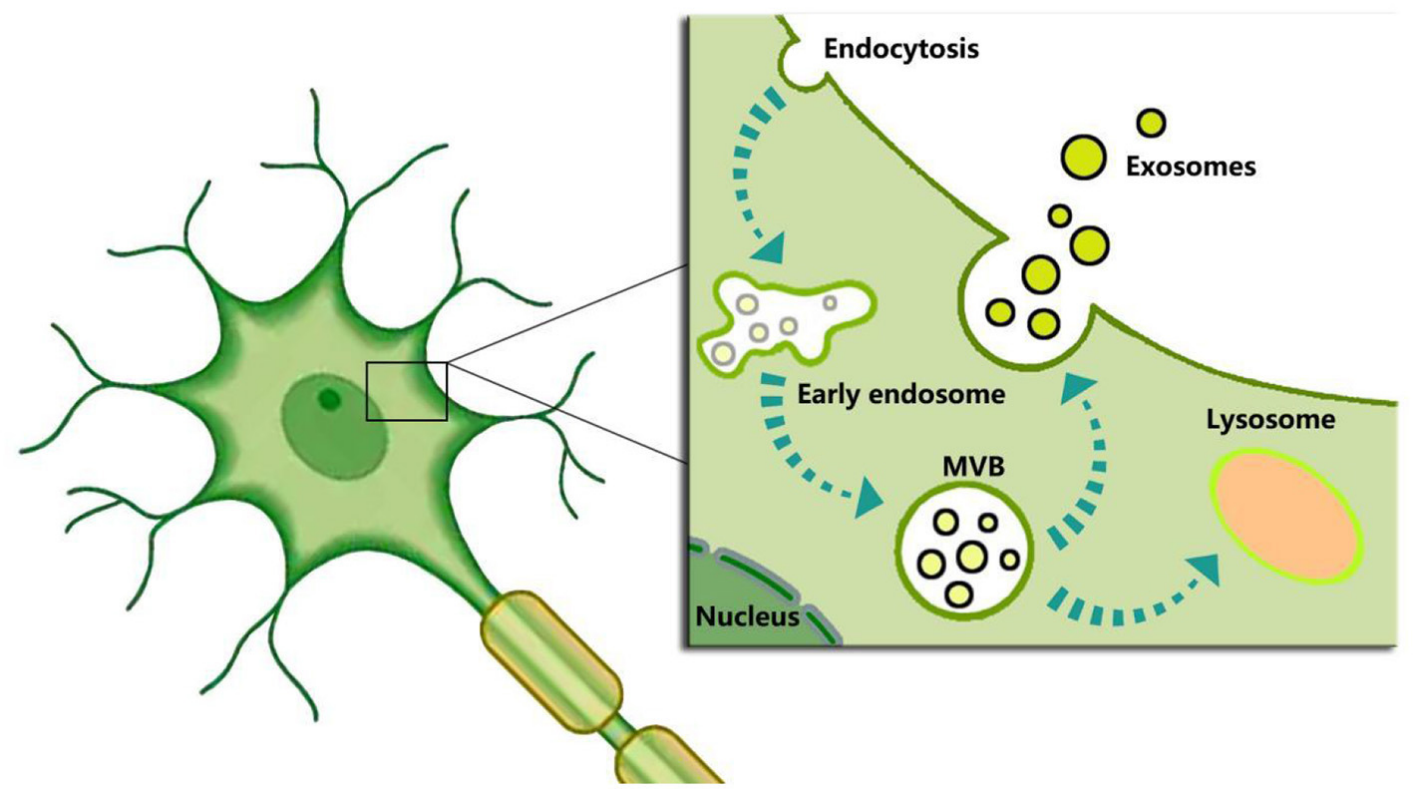
Schematic representation of exosome biogenesis. In short, exosome biogenesis begins with the inward budding of the plasma membrane to form endosomes. Then endosomes encapsulate nucleic acids, proteins, and other bioactive molecules to form intraluminal vesicles (ILVs) and multivesicular bodies (MVBs). Finally, the MVBs not fused by lysosomes are released into the extracellular environment to form “exosomes” after the fusion with the plasma membrane.

Given their specificity, feasibility, cost, and other considerations, the above predictive indicators for DEACMP have not yet gained expert consensus or widespread clinical use. As a result, there is still a need to explore new predictors for predicting DECAMP. The existing knowledge indicates that brain cells can secrete exosomes, which have become a hot field for evaluating brain function and diagnosing brain diseases. For example, neuronal-derived exosomes participate in the physiological and pathological processes of the brain, and changes in their content could provide valuable information for clinical diagnosis and disease evaluation of neurodegenerative diseases, as well as predict the occurrence of diseases at an early stage. Neuronal-derived exosomes could serve as potential biomarkers (105). Scholars have elucidated the process of exosome biogenesis, as illustrated in Figure 1, but the mechanisms behind the sorting of exosomal contents remain unclear. Changes in both intracellular and extracellular environments can influence the contents of exosomes and induce different biological functions. Indeed, intracellular hypoxia, ROS, and Ca^2+^ can significantly affect exosome biogenesis and their contents (76, 106, 107), while they play crucial roles in the pathogenesis of DEACMP.

Hypoxia promotes the release of exosomes and changes the composition of the exosomal contents, especially miRNAs (108). Chiang et al. harvested the exosomes from the rat cortical primary neuronal cells under normoxic or oxygen and glucose deprivation (OGD) conditions. They compared the exosomal miRNA expression levels in normoxic and OGD conditions using next-generation sequencing. They found that 45 exosomal miRNAs were significantly different in their expression levels. The expression levels of 18 exosomal miRNAs were significantly higher, but those in 27 exosomal miRNAs were lower than those of normoxic, respectively. These exosomal miRNAs may play a role in cellular survival or death processes, as well as neuronal signaling (109). In the upregulated exosomal miRNAs, miRNA-34a-5p and miR-10a-5p have caught our attention. Previous studies have found that increased miRNA-34a facilitates neuronal injury and death in ischemic brain injury (110), while miR-10a induces apoptosis and inflammatory responses in neurons by inhibiting the PI3K/Akt/mTOR pathway (111, 112). In contrast, in the downregulated exosomal miRNAs, miR-25-5p and miR-532-3p have caught our attention, due to their neuroprotective effects in ischemic brain injury (113, 127). CO can cause cerebral hypoxia after prolonged exposure, resulting in affecting the sorting mechanisms of exosomal miRNAs, and the above changed exosomal miRNA profiles may reflect the risk of DECAMP.

Oxidative stress influences exosome biogenesis through different pathways, showing both prompt or inhibit effects on the release of exosomes (114, 115). On one hand, ROS inhibits the release of calcium ions from lysosomes through the transient receptor potential mucin 1 (TRPML1) channel and blocks the fusion between multivesicular bodies (MVBs) and lysosomes (114). ROS also affects the function of normal lysosomes to form nonfunctional lysosomes by inhibiting the activity of mTOR, resulting in reduced degradation of MVBs (116), further upregulating exosomal release. On the other hand, ROS enhances the biogenesis of autophagosomes, and MVBs can be selectively degraded by autophagosomes, further downregulating exosomal release (117). In simple terms, low oxidative stress restrains MVBs from being degraded in lysosomes, thereby prompting the release of exosomes. In contrast, high oxidative stress promotes the degradation of MVBs by activating cellular autophagy, thereby inhibiting the release of exosomes (118). Indeed, these effect of oxidative stress also depend on donor cell type and extracellular stimulus conditions. In addition, oxidative stress may affect the sorting mechanisms of exosomal content, leading to changes in exosomal miRNA profiles. In turn, exosomal miRNAs can regulate various genes associated with oxidative stress. This crosstalk between exosomal miRNAs and oxidative stress plays a significant role in the pathophysiological process of neurodegenerative diseases (105). Several exosomal miRNAs have been associated with neurodegenerative disorders, such as exosomal miRNA-34a. It is well known that CO can promote the production of mitochondrial ROS, leading to intracellular oxidative stress in brain cells, that can influence the release and the molecular cargo of brain cell-derived exosomes after ACOP. Special exosomal miRNAs could be involved in the development of DEACMP and their changes in quantity can provide dynamic information for predicting DEACMP.

Intracellular Ca^2+^ also regulates exosome biogenesis, and increasing intracellular Ca^2+^ stimulates the release of exosomes (106). Hettiarachchi et al. found that CO stimulates the generation of NO and ROS, leading to the formation of peroxynitrite in brain tissue, aggravating oxidative stress, and disrupting neuronal Ca^2+^ homeostasis (119), which may inhibit the release of exosomes. Some scholars suggested that exosomes play a role as the bridge between the inflammasome and autophagy activation under CO exposure conditions (120).

Therefore, gaining an in-depth and comprehensive understanding of the role of exosomes in ACOP can clarify the pathogenesis of DECAMP and provide theoretical support for the research of exosomes as biomarkers to predict DECAMP. To date, there are few reports focused on the relationship between exosomes and DECAMP, as well as the potential use of exosomes as clinical indicators for DEACMP. This scarcity may stem from the blood or cerebrospinal fluid samples containing exosomes derived from various tissues or cells except brain cells. Existing isolation and purification methods of exosomes (e.g., ultracentrifugation, exosome isolation reagent, ultrafiltration, immunoprecipitation/affinity capture, etc.) do not effectively isolate large quantities of pure and specific brain cell-derived exosomes from in blood or cerebrospinal fluid samples. Hence, it is not easy to identify the exosomes needed to predict DECAMP under the current research conditions.

## Summary and outlook

DEACMP is a serious complication of ACOP that often shows poor therapeutic responses to current clinical treatment methods. Evaluating the risk of the development of DEACMP becomes very important. So far, some valuable predictors have emerged in clinical characteristics, laboratory indicators, neuroelectrophysiology, imaging examination, and genetic susceptibility. Recent research has confirmed that imaging examination (High cranial DWI signal within 24 h) and clinical characteristics (duration of CO >5.5 h) can serve as independent predictors of DEACMP (121). Some scholars highlighted the importance of the period of inability to walk in the acute stage and suggested that the clinical score of peak CK (U/L) and 40 × WALK (time for which walking was impossible during the acute stage of intoxication, days) is an early predictor for predicting DEACMP with 100% sensitivity and 82% specificity (122). More independent factors will also emerge in the future. Given the pathogenesis of DEACMP is diverse and complex, we need some new insights to explore the independent predictors. The studies on the unique role of exosomes in neurodegenerative diseases may offer new candidates to us. First, changes in both intracellular and extracellular environments of brain tissue after ACOP will influence the biogenesis and selection of contents in brain cell-derived exosomes, and exosomal contents may play roles in the pathogenesis of DEACMP, particularly miRNA. Identifying specific exosomal miRNA can reflect the development process of DEACMP. Second, brain cell-derived exosomes can cross the blood-brain barrier (BBB) and present in cerebrospinal fluid and blood circulation, facilitating us to collect them for serial determination about their amount and cargo. At last, CSF exosomal miRNAs can reflect brain pathophysiology while plasma exosomal miRNAs are not as easily degraded (123), which provides the samples to detect specific miRNA sequences using the ddPCR-based method. Based on this, exosomes have a potential advantage over currently identified predictors. Developing an effective isolation and purification method for wanted exosomes is a significant challenge in the current research on identifying specific exosomes for predicting DECAMP. Encouragingly, scholars have found that neuronal-derived exosome membranes are rich in the neuronal cell adhesion molecule and the L1 cell adhesion molecule (L1CAM). They utilized immunoprecipitation with biotinylated antibodies against neuronal surface markers to isolate neuronal-derived exosomes. This isolation method for separating will provide specific exosomes needed for DECAMP research (115, 124). With advancements in technology and increased research investment, we are optimistic that scholars will gain deeper insights into the role of exosomes in DECAMP and develop exosome-based detection methods for predicting DECAMP.
